# Noise-Aware Bayesian Optimization for Precision Improvement of Micro-Volume Liquid Handling in In Vitro Diagnostics

**DOI:** 10.3390/mi17101128

**Published:** 2026-09-28

**Authors:** Lihao Bai

**Affiliations:** Kaihe Biotechnology (Shanghai) Co., Ltd., Shanghai 200120, China; bailh@quaerolife.com

**Keywords:** microliter dispensing, in vitro diagnostics, Bayesian optimization, Gaussian process, heteroscedastic noise, coefficient of variation

## Abstract

Micro-volume dispensing precision is critical for in vitro diagnostic (IVD) analyzers. We present a noise-aware Bayesian optimization framework that minimizes the within-run coefficient of variation (CV) of a 50 μL dispensing process by optimizing five pump-control variables. Each setting was tested in five independent batches; the group mean CV was used as the response, and the squared group standard error was supplied to an automatic relevance determination Gaussian process as observation-noise variance. From 33 tested combinations, the lowest measured mean CV was 0.266% (SD, 0.044%). An engineering-rounded setting then achieved 0.270% (SD, 0.050%), an 81.8% relative reduction versus engineer-selected settings (1.480%, SD, 0.083%). In a retrospective surrogate-based replay, the noise-aware strategy reached CV < 1.5% in 2.8 ± 1.2 iterations, compared with 4.1 ± 2.0, 6.5 ± 3.4, and 11.3 ± 5.1 for homoscedastic GP, standard GP, and random search. Deionized water, diluted human serum, and 5% bovine serum albumin all yielded mean CVs below 0.45%. The method thus identified a repeatable low-CV operating region with a limited physical-experiment budget; prospective algorithmic comparisons and multi-instrument validation remain necessary.

## 1. Introduction

Microfluidic and automated liquid-handling technologies underpin many laboratory and point-of-care diagnostic workflows [1,2,3,4,5]. For automated liquid-handling systems, volumetric performance must be established by traceable measurement procedures and uncertainty-aware reporting [6]. In an IVD analyzer, a small dispensing deviation can propagate into the reagent-to-sample ratio and, ultimately, the analytical result. Precision is commonly summarized by the coefficient of variation (CV), but the observed CV is jointly affected by dispensing-pump speed and acceleration, priming-pump speed and acceleration, and the aspirated air-column volume. Because these variables interact through transient pressure, hydraulic resistance, and gas–liquid interfacial dynamics, a sufficiently accurate first-principles optimization model is difficult to establish. Engineering tuning therefore remains expensive and may not be repeatable when measurement variability is ignored.

Classical response-surface and population-based optimization methods can be effective when evaluations are inexpensive. Here, however, every objective value requires repeated physical dispensing and gravimetric measurement. A method must therefore learn from a small experimental budget while accounting for the fact that repeated batches at the same setting do not have constant dispersion. Treating CV as deterministic can make an incidental low observation appear optimal and can direct subsequent experiments toward a noise-induced pseudo-optimum.

Bayesian optimization (BO) is designed for expensive black-box functions and uses a probabilistic surrogate plus an acquisition function to balance exploration and exploitation [7,8,9,10,11]. Gaussian-process (GP) surrogates can incorporate known observation variances and can also be extended to input-dependent noise [8,12,13,14]. This is relevant to dispensing experiments because a high-CV operating region may also exhibit greater between-batch dispersion than a stable low-CV region. Although BO is now widely used in adaptive experimental design, the use of replicate-derived, group-specific observation variances for tuning microliter-scale IVD fluidics remains insufficiently documented.

Microvalves, micropumps, flow sensors, and non-contact dispensing structures have been developed for precise microvolume delivery [15,16,17,18,19], while adaptive experimentation offers a general route to more autonomous laboratory optimization [20,21]. Building on these developments, this study makes three contributions: (i) it defines a five-variable optimization problem on a physical IVD dispensing platform; (ii) it uses five independent batches at each setting to estimate a group-specific standard error and supplies its square to the GP as observation-noise variance; and (iii) it evaluates the selected operating region by independent comparison with engineer-selected settings and by preliminary multi-matrix testing. The aim is to determine whether a low-CV region can be located with 33 physical parameter evaluations, not to claim universal superiority over all optimization strategies.

## 2. Noise-Aware Bayesian Optimization for the Dispensing System

The dispensing system coordinates a sampling needle, pumps, and valves to aspirate a precise amount of biological sample (e.g., blood, urine, or semen) from a sample cup and dispense it into a reaction cup. Dispensed-volume accuracy has a direct effect on IVD results. A representative fluidic system is shown in Figure 1. Before aspirating the sample, the sampling needle draws in an air column that separates the sample from the system liquid. Liquid-level detection is then performed, after which a stepper motor drives plunger pump R01 to generate the vacuum required for aspiration. Finally, the sampling arm rotates the needle to the reaction cup and dispenses the sample.

The aspiration–dispensing–retraction cycle is governed by five key control variables: dispensing-pump speed, dispensing acceleration, priming-pump speed, priming acceleration, and aspirated air-column volume. These variables do not act independently; instead, they interact through transient pressure, hydraulic resistance, and liquid–gas interfacial behavior. Their combined effect changes the actual volume delivered in each operation and is ultimately reflected in the CV of dispensing precision.

For the intended application, the dispensing CV is required to remain below 1.5%. CV fluctuated even when the programmed settings were unchanged. Plausible contributors include gravimetric uncertainty, pump vibration, microbubble dynamics, and uncontrolled changes in liquid properties; these contributors were not separately instrumented in the present study. The observations were modeled as conditionally Gaussian around an unknown response surface. An automatic relevance determination (ARD) squared-exponential kernel assigned an independent length scale to each input. A diagonal matrix Σ was added to the kernel matrix K, with each entry defined by the squared standard error of the corresponding group. Thus, observations from settings with larger estimated uncertainty exerted less influence on the fitted response surface. A homoscedastic GP was retained only as a retrospective surrogate-model comparator.

## 3. Theoretical Analysis of Factors Affecting Dispensing Precision

### 3.1. Quantitative Effect of Air-Column Compressibility on Dispensing Precision

As shown in Figure 2 below, before liquid aspiration, the system draws an air column of volume V_0_ to separate the sample from the system liquid. The air column is exposed to a continuously varying pressure field inside the tubing. Because gases are far more compressible than liquids, the air column’s volume changes with pressure and alters the volume ultimately dispensed into the reaction cup.

For an order-of-magnitude sensitivity calculation, the gas compression is approximated as isothermal. This assumption requires experimental verification from pressure-transient data. From the ideal-gas law, the air-column volume change caused by a small pressure fluctuation is given by Equations (1) and (2). In the present system, the air-column range is 5–50 μL. Illustrative values of V_0_ = 20 μL and p_0_ = 101.325 kPa are used below.

(1) $$\frac{dV}{dp}=-\frac{p_{0}V_{0}}{p^{2}}\approx -\frac{V_{0}}{p_{0}}\;\;(p\approx p_{0})$$

(2) $$\delta V_{air}\approx -V_{0}\frac{\delta p}{p_{0}}$$

Thus, a pressure fluctuation of only 1 kPa changes the volume of a 20 μL air column by approximately 0.2 μL.

During aspiration and dispensing, the pressure in the tube is determined by the motion of the plunger pump rather than being constant. Neglecting gravity and treating the liquid as an incompressible Newtonian fluid in laminar flow through a circular tube, the pressure gradient can be described by the Hagen–Poiseuille equation:

(3) $$\Delta p_{pipe}=\frac{128\mu L}{\pi D^{4}}Q$$

where μ = 1.0 × 10^−3^ Pa·s is the dynamic viscosity of water; L = 0.5 m is the characteristic tube length from the sampling needle to the pump valve; D = 0.8 mm is the tube inner diameter; and Q is the volumetric flow rate, which is directly related to pump speed (Q = 60 μL/s is used for illustration).

(4) $$\Delta p_{pipe}=\frac{128\times 1.0\times 10^{-3}\times 0.5}{\pi \times (8\times 10^{-4}{)}^{4}}\times 6\times 10^{-8}\approx 7.4\;kPa$$

Equation (4) gives the distributed pressure loss in the tube. Local losses also occur at the narrowed needle tip and valve orifices. Using these values, of a local pressure loss K ≈ 0.8, a needle-tip inner diameter of 0.5 mm, and a local velocity v = 0.31 m/s, gives

(5) $$\Delta p_{local}=K\frac{\rho v^{2}}{2}$$

(6) $$\Delta p_{local}=0.8\times \frac{1000\times {\left(0.31\right)}^{2}}{2}\approx 38\;Pa$$

The third and largest component is the pressure impulse generated by pump acceleration and deceleration. As shown in Equation (7), accelerating the liquid column produces an additional pressure:

(7) $$\Delta p_{accel}=\rho La$$

(8) $$a=\frac{4\dot{Q}}{\pi D^{2}}$$

For a dispensing acceleration corresponding to a linear acceleration of approximately 10.2 m/s^2^:

(9) $${\Delta p}_{accel}=1000\times 0.5\times 10.2\approx 5.1\;kPa$$

Combining Equations (4), (6) and (9), the maximum pressure difference over a complete cycle is approximately

(10) $$\Delta p_{max}\approx \Delta p_{pipe}+\Delta p_{local}+\Delta p_{accel}\approx 7.4+0.038+5.1\approx 12.5\;kPa$$

The corresponding air-column volume fluctuation is

(11) $$V_{air}=V_{0}\frac{\Delta p_{max}}{p_{0}}=20\times \frac{12.5\times 10^{3}}{101325}\approx 2.47\;\mu L$$

Let ηt denote the fraction of air-column volume variation that appears as delivered-volume deviation. Using ηt = 0.7 as an illustrative engineering assumption—not an independently calibrated coefficient—the corresponding error estimate is

(12) $$\delta V_{sample}\approx 0.7\times \delta V_{air}\approx 1.73\;\mu L$$

Equation (12) is a scaling argument rather than a validated prediction. It suggests that pump speed, acceleration, and air-column volume should be optimized jointly, but direct pressure/volume transient measurements are required to calibrate ηt and to test the assumed flow and thermal regimes quantitatively.

### 3.2. Critical Conditions for High-Speed Aspiration-Induced Vortices and Air Entrainment

During aspiration, liquid at the needle tip accelerates from rest to the internal tube velocity. The free surface is governed by competition between inertia, which deforms or disrupts the interface, and surface tension, which tends to preserve it. The relative strengths of these effects are described by the Weber number:

(13) $$We=\frac{\rho u^{2}D}{\gamma }$$

where ρ = 1 × 10^3^ kg/m^3^ is the liquid density, D = 0.5 mm is the needle-tip inner diameter, and γ = 0.073 N/m is the surface tension of water. When the dispensing-pump speed corresponds to a tip velocity u = 0.2 m/s (a volumetric flow rate of approximately 40 μL/s),

(14) $$We=\frac{1000\times (0.2{)}^{2}\times 0.5\times 10^{-3}}{0.073}\approx 0.27$$

As shown in Equation (14), We < 1; surface tension remains dominant, the interface remains intact, and air is not entrained. If the velocity is increased to u = 0.5 m/s,

(15) $$We=\frac{1000\times (0.5{)}^{2}\times 0.5\times 10^{-3}}{0.073}\approx 1.71$$

In this case, We > 1, inertia overcomes surface tension, the free surface is disrupted, and a vortex entrains air to form a discontinuous gas column. The aspirated volume consequently deviates from the target. Even when the steady-state velocity is moderate, excessive acceleration can drive the interface into a transient high-shear state. Dispensing-pump speed and acceleration must therefore be constrained jointly to prevent vortex formation and air entrainment.

### 3.3. Influence of Dispensing Needle, Tubing, and Connector Geometry

The geometry of the dispensing flow path—needle inner diameter, tubing inner diameter and length, and connector dead volume—can also affect dispensing precision through flow resistance, backpressure, dead volume, and bubble entrapment. For a cylindrical tube, the laminar pressure drop is governed by the Hagen–Poiseuille relation,

(16) $$\Delta P\propto \frac{L}{r^{4}}$$

where L is the tube length and r is the inner radius. Thus, even small reductions in inner diameter can substantially increase backpressure and flow instability; conversely, very wide tubing can increase total system volume and syringe compliance, which may degrade precision. In the present configuration (needle inner diameter 0.5 mm, tubing inner diameter 0.8 mm, tubing length ≈ 0.5 m, and minimal connector dead volume), the estimated pressure drop at the operating flow rates is expected to be small relative to the pressure impulses generated by pump acceleration (see Section 3.1). Moreover, a narrow needle tip increases the local velocity and can promote air entrainment when the Weber number approaches or exceeds unity (Section 3.2). Connector dead volume can trap residual liquid or micro-bubbles, contributing to carryover and between-dispense variability. Within the ranges available for this platform, the geometric contribution to CV is expected to be secondary to the five optimized pump-control variables. A dedicated experimental study that systematically varies needle size, tubing dimensions, and connector geometry is left for future work. 

## 4. Experimental Platform and Methods

### 4.1. Platform, Instrumentation, Materials, and Control Variables

Experiments were conducted on the fluidic module of an IVD analyzer. The dispensing and priming pumps had full-scale capacities of 100 and 1000 μL, respectively, and were driven by stepper motors operated at 16 microsteps and 2000 steps per full-scale stroke, corresponding to nominal displacement resolutions of 0.05 and 0.5 μL per step. The sampling needle had an inner diameter of 0.5 mm and an outer diameter of 0.8 mm. The polytetrafluoroethylene tubing had an inner diameter of 0.8 mm and a length of approximately 0.5 m. Dispensing-pump speed and acceleration, priming-pump speed and acceleration, and aspirated air-column volume were selected as optimization variables; their search ranges were determined from hardware limits and preliminary experiments (Table 1).

The experimental platform is shown in Figure 3. The dispensed mass was measured using a Mettler Toledo XPE56C analytical balance (52 g maximum capacity; 0.001 mg readability, Mettler-Toledo GmbH, Greifensee, Switzerland), with the internal automatic calibration routine performed daily. The balance was installed on a marble anti-vibration table and enclosed by an acrylic draft shield to reduce vibration, airflow, and evaporation effects. Laboratory temperature and relative humidity were maintained at 25 ± 0.5 °C and 45 ± 5%, respectively, and monitored continuously using a Testo 608-H1 thermo-hygrometer (Testo SE & Co. KGaA, Lenzkirch, Germany). All test liquids were equilibrated in the laboratory for at least 30 min before use. The main optimization used deionized water with a resistivity of at least 18.2 MΩ·cm and an assumed density of 0.998 g/mL at 25 °C. Gravimetric procedures were organized with reference to the traceability and uncertainty-reporting principles in ISO 23783-2:2022 [6].

### 4.2. Precision Metric and Repeated-Measurement Design

Within-run CV was used as the precision metric. For each parameter combination, the system dispensed 20 wells at a target volume of 50 μL. The first well was predefined as a conditioning/rinsing dispense and excluded from the analysis; the remaining 19 wells constituted one batch. As illustrated in Figure 4, delivered mass was measured gravimetrically and converted to volume using a density of 0.998 g/mL at 25 °C. The within-batch mean volume and sample standard deviation s were then calculated, and CV was defined as

(17) $$CV=\frac{s}{\bar{V}}\times 100\%$$

Fifteen initial combinations were generated by Latin-hypercube sampling with a Mersenne Twister random seed of 42. Each combination was evaluated in five independent batches, producing five within-run CV observations. Their mean was the response supplied to the surrogate model, and their standard error quantified the uncertainty of that group mean. The optimization target was CV < 1.5%. This threshold was established during instrument development based on accumulated clinical experience with similar IVD analyzers, where a within-run CV above this level could materially affect reagent-to-sample ratio accuracy and assay reproducibility; it serves as the practical acceptance limit for this dispensing step. The precision requirement adopted for the intended microliter-scale dispensing application.

The standard error used to quantify observation noise was calculated as follows:

(18) $$SE_{i}=\frac{s_{i}}{\sqrt{m}}$$

The group-specific standard error in Equation (18) was treated as the uncertainty of the observed group mean; its square was supplied as the corresponding diagonal observation-noise variance in the GP.

### 4.3. Noise-Aware Gaussian-Process Model and Sequential Search

The Bayesian optimization framework consisted of a GP surrogate model and an expected-improvement (EI) acquisition function [7,8,9,10,11,12,13,14].

A zero-mean GP was used with an ARD squared-exponential covariance function:

(19) $$k(x,x^{\prime })=\sigma_{f}^{2}exp\left(-\frac{1}{2}\sum_{i=1}^{5}\frac{(x_{i}-{x_{i}}^{\prime }{)}^{2}}{l_{i}^{2}}\right)$$

where $\sigma_{f}^{2}$ is the signal variance and $l_{i}$ is the length scale of input dimension *i*. Independent length scales allow the fitted model to represent differences in parameter sensitivity.

To account for observation noise, the standard error estimated from the five batch-level CVs at each setting was embedded explicitly in the covariance matrix. For N tested parameter combinations, Σ = diag(SE1^2^, SE2^2^, …, SEN^2^) was added to K. This is a plug-in estimate of heteroscedastic observation uncertainty rather than a jointly learned noise process. For an arbitrary candidate point x*, the GP posterior mean and variance were calculated as

(20) $$\mu (x_{*})=k_{*}^{⊤}(K+\Sigma {)}^{-1}\bar{y}$$

(21) $$\sigma^{2}(x_{*})=k(x_{*},x_{*})-k_{*}^{⊤}(K+\Sigma {)}^{-1}k_{*}$$

where k* is the covariance vector between x* and all training points, and $\bar{y}$ is the vector of observed mean CV values. 

EI was used to balance the exploration of uncertain regions with the exploitation of regions with a low predicted mean. Relative to the best observed objective value fmin, EI was calculated as

(22) $$EI\left(x\right)=\left(f_{min}-\mu \left(x\right)\right)\Phi \left(Z\right)+\sigma \left(x\right)\phi \left(Z\right),\;\;Z=\frac{f_{min}-\mu (x)}{\sigma (x)}$$

where Φ and φ are the cumulative distribution function and probability density function of the standard normal distribution, respectively. The EI exploration parameter was ξ = 0. At each iteration, 5000 candidate points were drawn uniformly from the bounded parameter space, EI was evaluated for every candidate, and the point with the largest EI was selected for the next experiment. The candidate count was selected in preliminary testing because further increases changed the recommended point by less than 0.5% of each variable range; one acquisition-function search required approximately 0.6 s on a single CPU core.

Figure 5 summarizes the sequential workflow. All five inputs were min–max scaled to [0, 1], whereas CV remained on its original percentage scale. The zero-mean ARD squared-exponential GP was implemented in MATLAB (v2022a) with the Statistics and Machine Learning Toolbox (fitrgp) and a custom BO routine. Kernel hyperparameters were obtained by maximizing the log marginal likelihood, while each group-specific SE^2^ was fixed as known observation-noise variance rather than estimated by the model. Each proposed point was evaluated in five physical batches and appended to the dataset. Sequential testing was stopped after the prespecified 18 iterations; at that point, the maximum EI was below 1 × 10^−3^. The final rounded setting underwent independent comparison and preliminary multi-matrix testing.

### 4.4. Statistical Analysis and Model Assessment

Model prediction was assessed by leave-one-out cross-validation (LOOCV) across the 33 tested parameter combinations, with root-mean-square error (RMSE) and mean absolute error (MAE) calculated on the original CV-percentage scale. Parameter stability was examined by 1000 bootstrap resamples of the 33 groups: an ARD GP was refitted to each resample and the resulting optimum was recorded to form empirical 95% intervals. Spearman rank correlations assessed monotonic associations between each control variable and group mean CV. The optimized and engineer-selected validation groups were compared using an independent-samples *t* test, and a Brown–Forsythe test assessed the equality of their CV dispersions [22,23,24]. Statistical significance was evaluated at α = 0.05. Analyses were implemented in MATLAB R2022a using a Mersenne Twister seed of 42, where random resampling was required.

## 5. Results and Discussion

### 5.1. Optimization Trajectory and Best Measured Designs

After the GP was initialized with 15 designs, the noise-aware Bayesian optimization algorithm recommended 18 additional parameter combinations. In total, 33 combinations were tested, with five independent batches per combination. Figure 6 shows the normalized levels of the five fluidic-control variables and their associated CV values. In the upper heat map, red and blue denote relatively high and low parameter levels, respectively. In the lower panel, gray points represent the five individual batch CVs, while blue points and error bars represent the group mean and standard deviation. The mean CV and its dispersion varied markedly across combinations. Groups 3, 10, 19, and 22 produced relatively high mean CVs and, in some cases, substantial between-batch dispersion. From Group 26 onward, all mean CVs were below the 1.5% target. Group 27 achieved the lowest mean CV, 0.266%, with a standard deviation of 0.044%; several subsequent groups also remained at low CV levels, which is consistent with a low-CV region rather than one isolated low observation.

Within the evaluated design set, low-CV experiments late in the optimization generally combined a high dispensing-pump speed, a relatively high priming-pump speed, a small air-column volume, and low-to-moderate pump accelerations, indicating substantial parameter interactions. The dispersion of repeated measurements also changed across parameter combinations, suggesting parameter-dependent, heteroscedastic observation noise. Because all five variables changed simultaneously, Figure 6 illustrates joint trends rather than the causal effect of any single variable; the ARD length scales were therefore treated only as an exploratory model-sensitivity summary.

### 5.2. Parameter Sensitivity

The inverse ARD length-scale ranking is shown in Figure 7. Dispensing-pump speed had the largest modeled sensitivity, followed by dispensing acceleration, priming acceleration, air-column volume, and priming-pump speed. Spearman analysis was consistent with a strong negative monotonic association between dispensing-pump speed and group mean CV (ρ = −0.68, *p* < 0.001) and a weaker negative association for dispensing acceleration (ρ = −0.31, *p* < 0.05); the other three associations were not statistically significant. Neither the correlations nor the ARD ranking should be interpreted as causal variance decomposition, because all variables were changed jointly and the model sensitivities depend on scaling, kernel specification, sampling density, and correlations among tested settings.

The visibly unequal error-bar magnitudes across the 33 settings are consistent with parameter-dependent dispersion and motivate the use of group-specific observation-noise variances. This interpretation remains descriptive because each variance was estimated from only five batches and no separate prospective noise-model validation was performed.

### 5.3. Convergence and Surrogate-Model Assessment

The 18 sequential trials progressively reduced the best observed CV below the 1.5% target (Figure 8). The best-so-far CV fell below the target after relatively few evaluations, decreased sharply from approximately 1.4% to 0.8% near Experiment 25, and ultimately approached 0.3%. The curve plateaued after Experiment 27, which is consistent with the algorithm having located a low-CV region among the sampled settings; it does not prove global convergence.

Across 33 parameter combinations and 165 batch experiments, the plotted best single-batch CV decreased from approximately 0.82% in the initial stage to approximately 0.20%. A practical parameter combination in the selected low-CV region was a dispensing-pump speed of 120 μL/s, dispensing acceleration of 580 μL/s^2^, priming-pump speed of 340 μL/s, priming acceleration of 510 μL/s^2^, and an air-column volume of 10 μL. This rounded combination was subsequently evaluated in independent validation experiments.

Across the 33 tested combinations, LOOCV yielded RMSE = 0.38 percentage points and MAE = 0.29 percentage points. In 1000 bootstrap resamples, the empirical 95% intervals for selected settings were 115–126 μL/s for dispensing-pump speed, 8–13 μL for aspirated air-column volume, and 320–360 μL/s for priming-pump speed. These intervals indicate that the recovered low-CV region was comparatively stable along three practically important dimensions; they do not quantify uncertainty for unreported variables or prove a unique global optimum.

### 5.4. Retrospective Same-Budget Offline Replay

To compare search behavior at an equal budget without conducting additional physical experiments, a robust GP fitted to all 33 measured combinations was treated as a surrogate ground-truth response surface. Four strategies were replayed from the same 15 initial Latin-hypercube points for 18 simulated rounds: (i) the proposed noise-aware GP using group-specific SE^2^ values; (ii) a homoscedastic GP using the median SE as a global noise level; (iii) a standard GP with noise estimated automatically by fitrgp; and (iv) uniform random search. Each replay was repeated 50 times. Because outcomes were generated from a fitted surrogate rather than new physical measurements, this analysis is a retrospective simulation and not a prospective head-to-head experiment.

The noise-aware replay reached the 1.5% threshold in fewer simulated iterations and achieved a lower terminal best CV than the three comparators (Table 2). These results are consistent with a benefit from using replicate-derived observation uncertainty on the fitted surface; however, they may inherit structural assumptions and bias from that same surrogate and therefore cannot establish an independent experimental contribution of the noise model.

## 6. Experimental Validation

The engineering applicability of the selected parameter combination was evaluated through an independent precision comparison and a preliminary multi-matrix experiment.

### 6.1. Precision Validation

The practical benefit of the selected setting was assessed against an engineer-selected parameter combination under identical conditions (same matrix, ambient temperature, day, and balance). The selected optimized combination—dispensing-pump speed 120 μL/s, dispensing acceleration 580 μL/s^2^, priming-pump speed 340 μL/s, priming acceleration 510 μL/s^2^, and air-column volume 10 μL—was evaluated in 10 independent dispensing batches at a target volume of 50 μL. Within-run CV was calculated after excluding the first well. The distributions are shown in Figure 9.

The engineering-rounded combination produced a mean within-run CV of 0.270% across the 10 validation batches (SD, 0.050%; SE, 0.016%; 95% CI, 0.234–0.306%). Every batch was below 0.40% and below the 1.5% application target.

The engineer-selected setting produced a mean CV of 1.480% (SD, 0.083%; SE, 0.026%; 95% CI, 1.421–1.539%). The absolute difference was 1.210 percentage points, corresponding to an 81.8% relative reduction for the optimized setting. The source reports *p* < 0.001 for the independent-samples *t* test and *p* < 0.05 for the Brown–Forsythe comparison of dispersion. 

### 6.2. Multi-Matrix Robustness Validation

As shown in Figure 10 below, to examine robustness to clinically relevant matrix variation, deionized water, human serum diluted 1:10 with phosphate-buffered saline (PBS), and 5% bovine serum albumin (BSA) solution were each tested in five independent batches using the selected parameters. Mean within-run CVs were 0.270% for water, 0.350% for diluted serum, and 0.440% for BSA solution, all below the 1.5% design target. No formal between-matrix inference was specified. The numerical increase for serum and BSA could reflect differences in viscosity or interfacial properties, but those properties were not measured and only five batches per matrix were tested. These results are therefore preliminary evidence of cross-matrix applicability, not a demonstration of equivalence or clinical-sample robustness.

## 7. Conclusions

This study formulated microliter-scale IVD dispensing as a five-variable, noisy black-box optimization problem. Five independent batches at each setting were used to estimate both a group mean CV and a setting-specific standard error, and the squared standard error was supplied to an ARD GP with EI. Fifteen initial designs and 18 sequential trials evaluated 33 parameter combinations. The best measured group mean was 0.266%, and the independently tested engineering-rounded setting achieved a mean CV of 0.270% (SD, 0.050%), compared with 1.480% (SD, 0.083%) for engineer-selected settings (reported *p* < 0.001), corresponding to an 81.8% relative reduction. All tested matrices remained below the 1.5% application target.

LOOCV yielded RMSE and MAE values of 0.38 and 0.29 percentage points, respectively. Bootstrap analysis localized the selected region, with 95% intervals of 115–126 μL/s for dispensing-pump speed, 8–13 μL for air-column volume, and 320–360 μL/s for priming-pump speed. A retrospective same-budget replay on a surrogate ground-truth surface favored the noise-aware strategy over three comparators; because those trajectories were simulated rather than prospectively run on the physical system, they support method plausibility but do not establish experimental algorithmic superiority. The ARD ranking is likewise model-dependent and should not be interpreted as causal evidence for individual fluidic mechanisms.

The main limitations are the absence of a same-budget prospective comparison with alternative optimizers, the use of a single instrument and dispensing volume, the evaluation of only five batches per matrix, and the need to document the provenance and ethical governance of the diluted human-serum material. The 0.7 transfer coefficient used in the Section 3 was illustrative rather than calibrated. Future work should release the complete batch- and well-level dataset and analysis code, compare algorithms over repeated random seeds, measure pressure/flow transients, validate across instruments, volumes, days, operators, and appropriately governed clinical samples, and conduct systematic experimental characterization of needle, tubing, and connector geometry effects. This remains an open question for interested researchers to address in future studies.

## Figures and Tables

**Figure 1:**
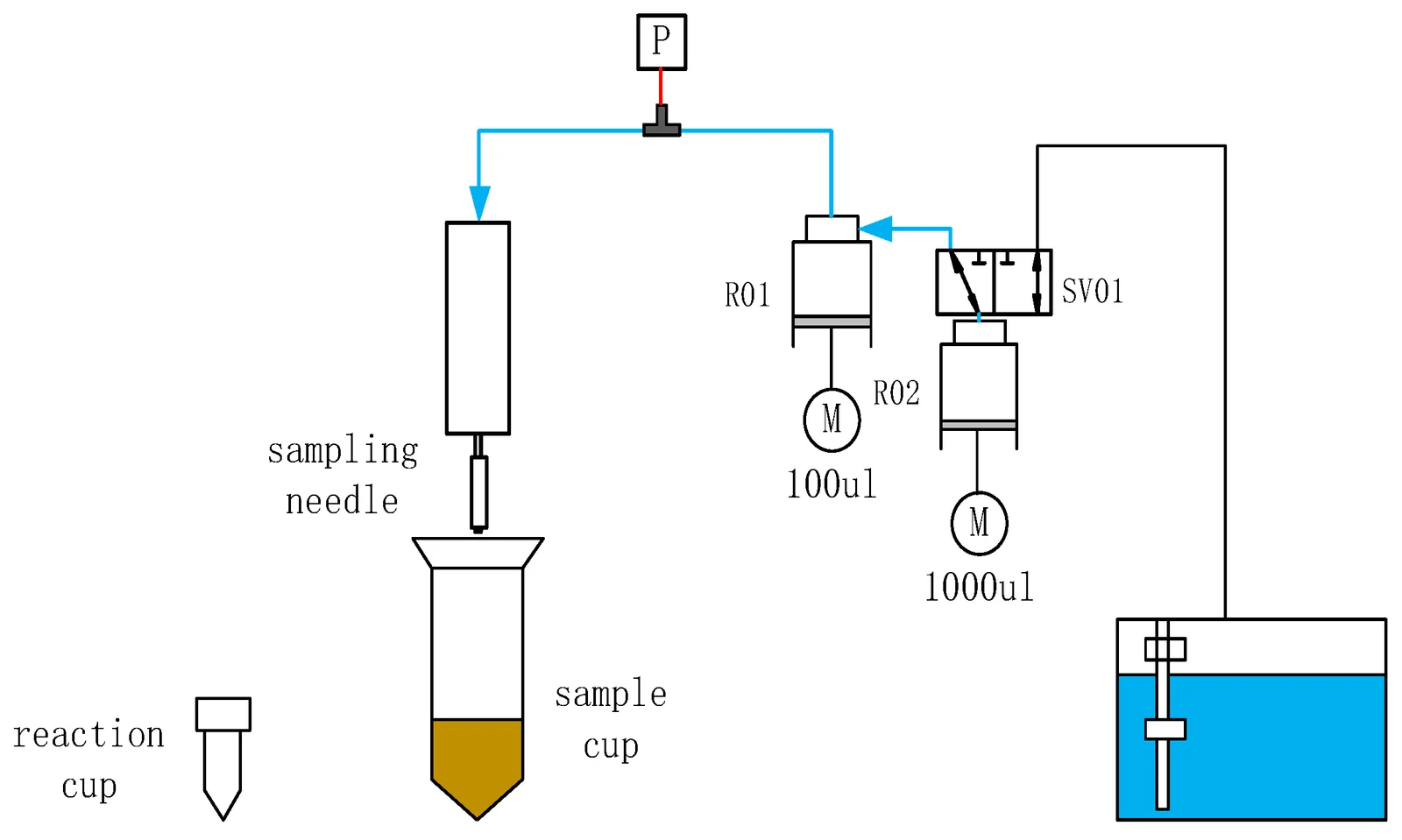
Fluidic schematic of the dispensing system.

**Figure 2:**
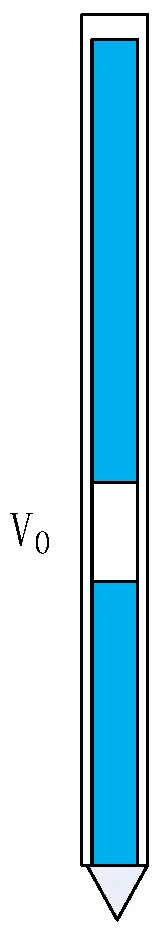
Air-column configuration during dispensing.

**Figure 3:**
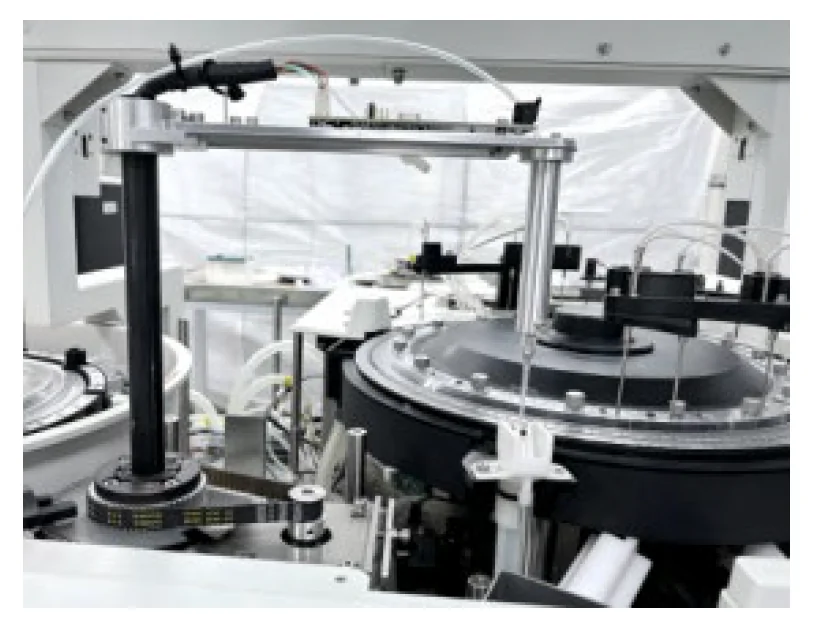
Experimental dispensing platform.

**Figure 4:**
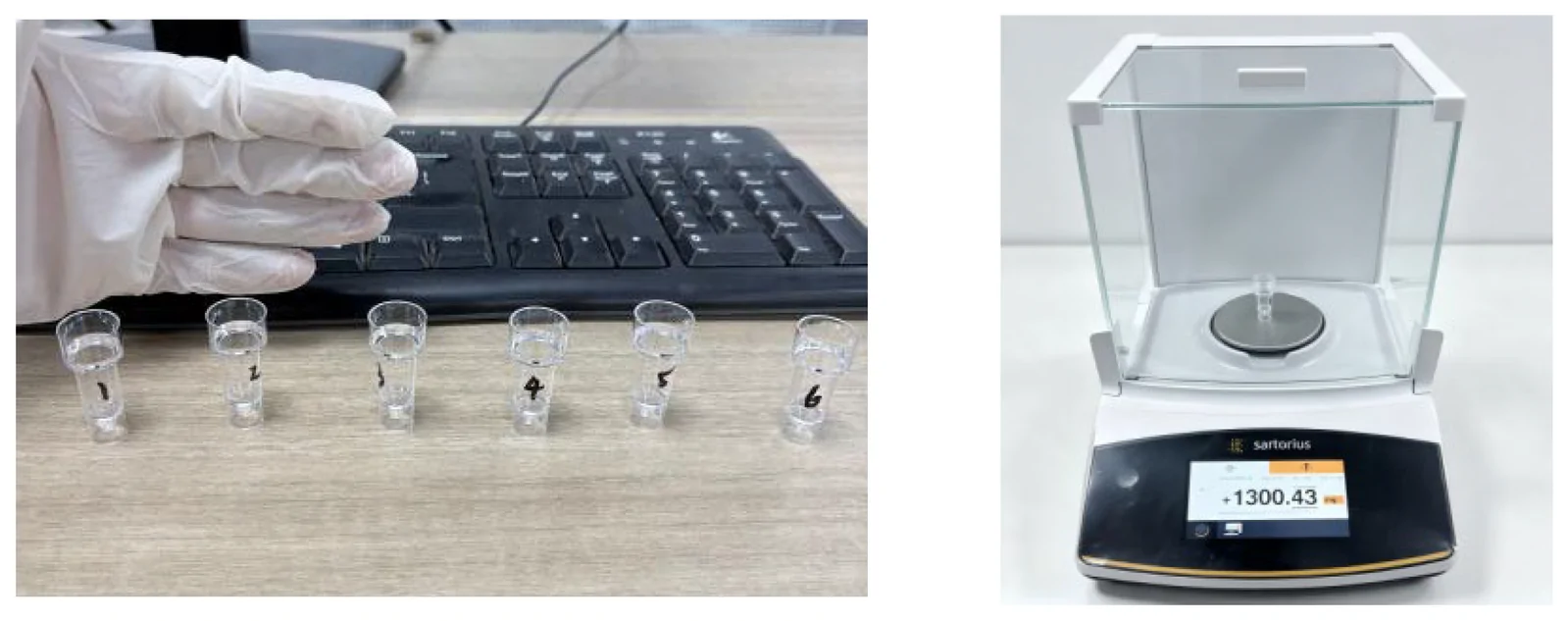
Tared sample cups and gravimetric measurement.

**Figure 5:**
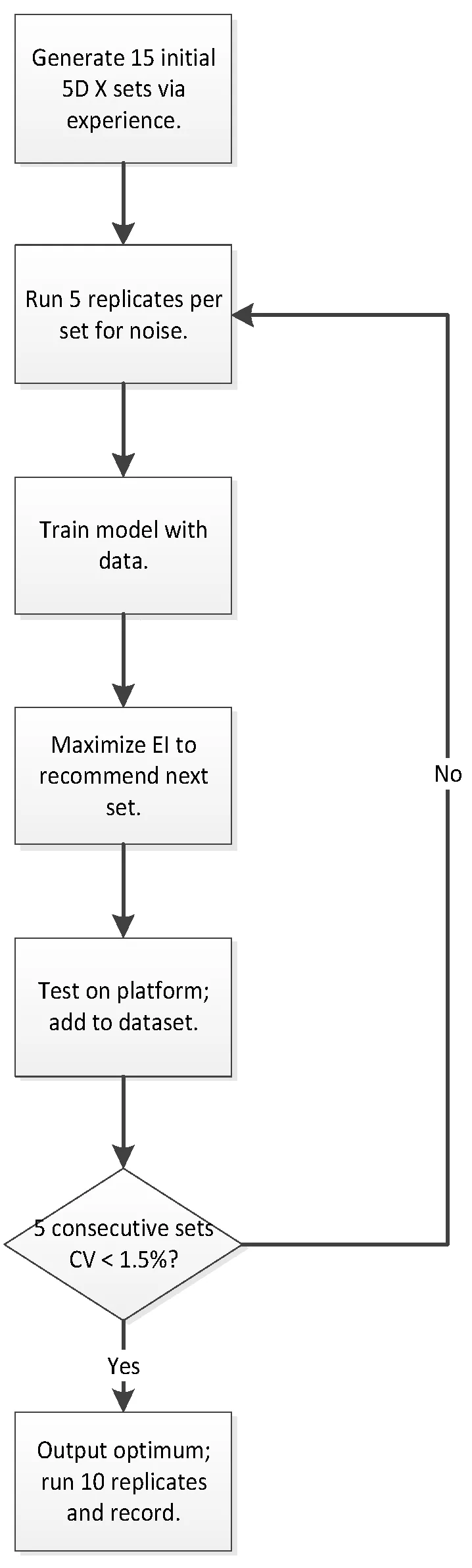
Workflow of the noise-aware Bayesian optimization procedure.

**Figure 6:**
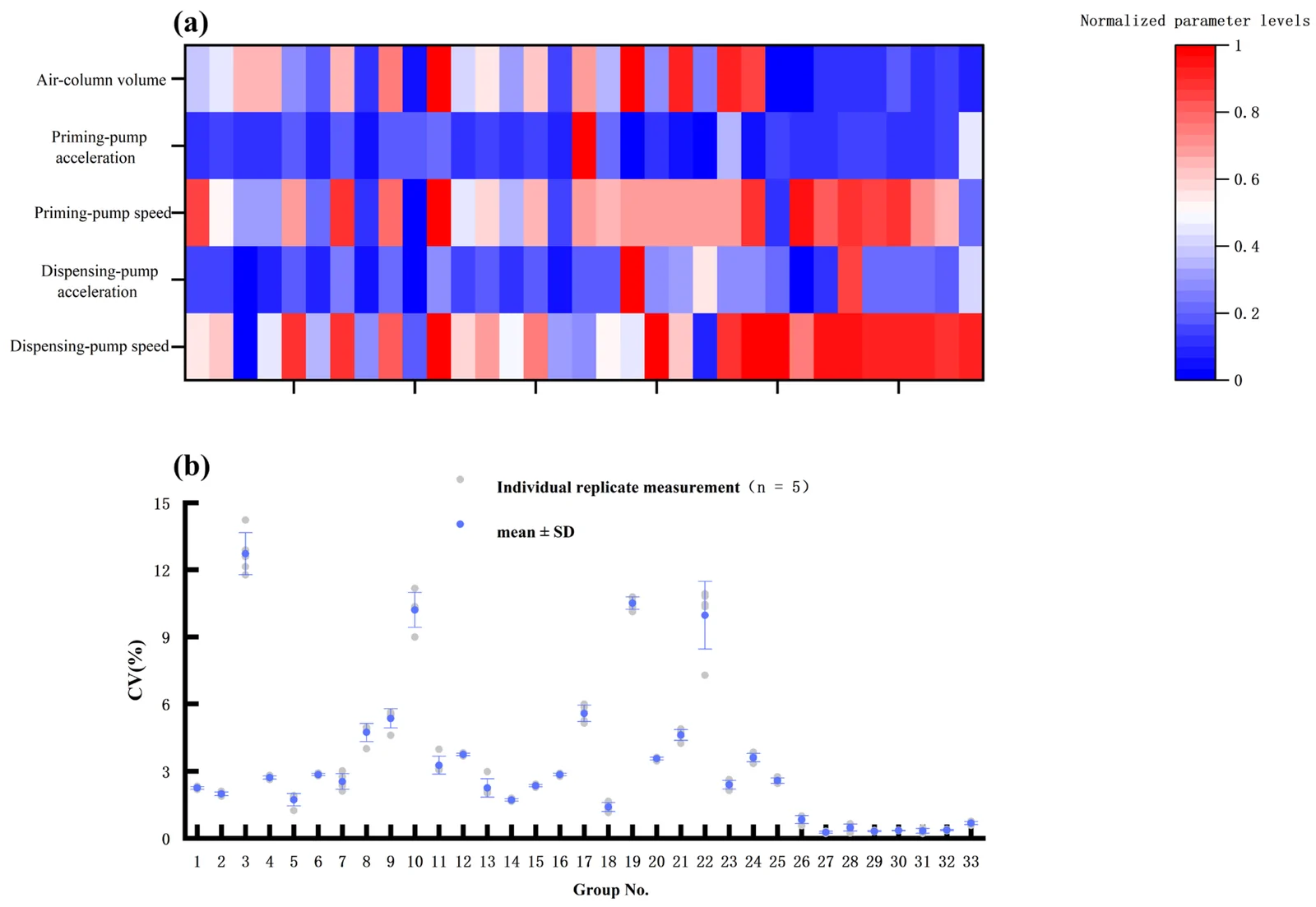
(**a**) Normalized levels of five control parameters across the 33 tested combinations; (**b**) individual batch CV measurements and group mean ± SD for each combination (five batches per group).

**Figure 7:**
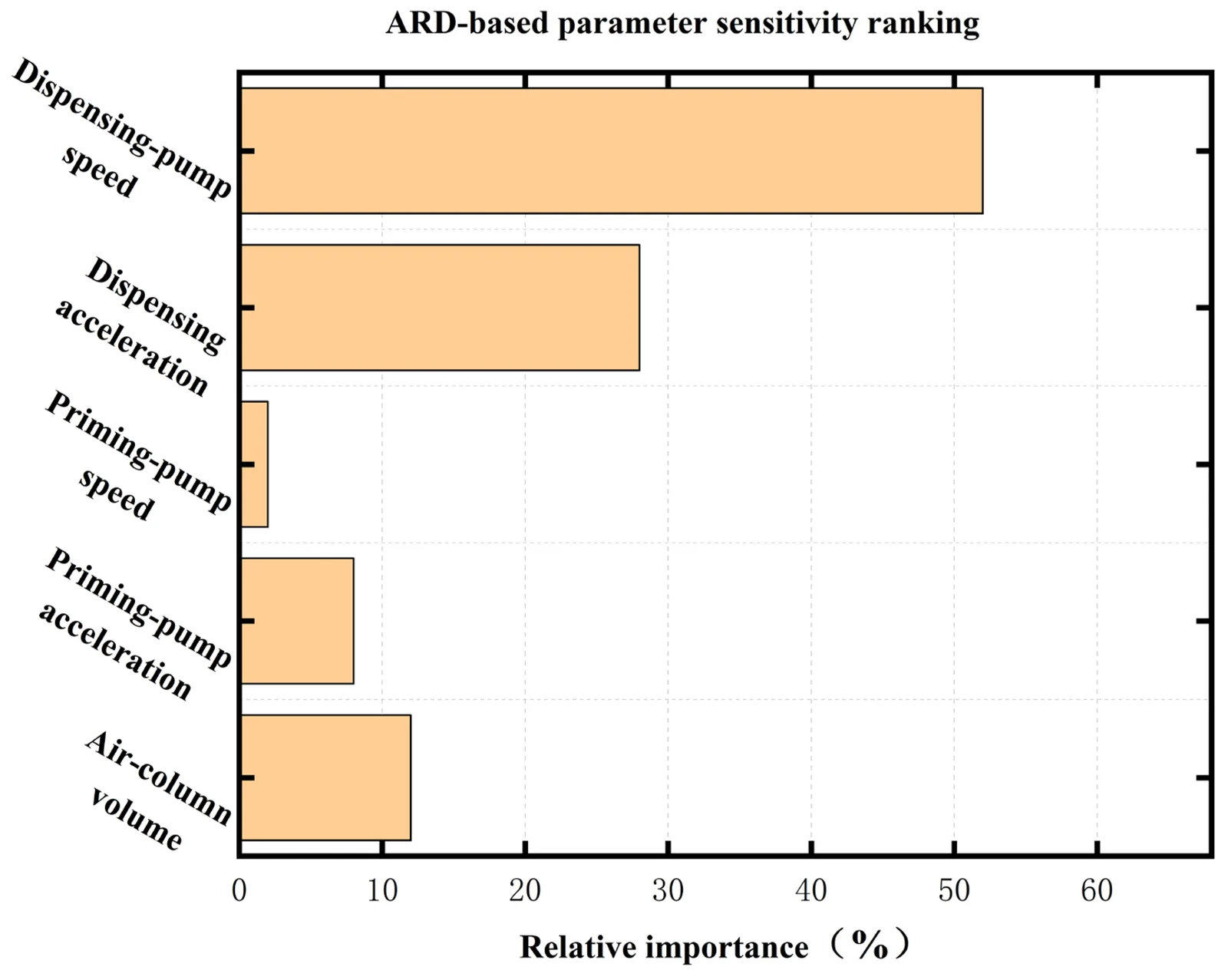
ARD-based sensitivity ranking of the five control parameters.

**Figure 8:**
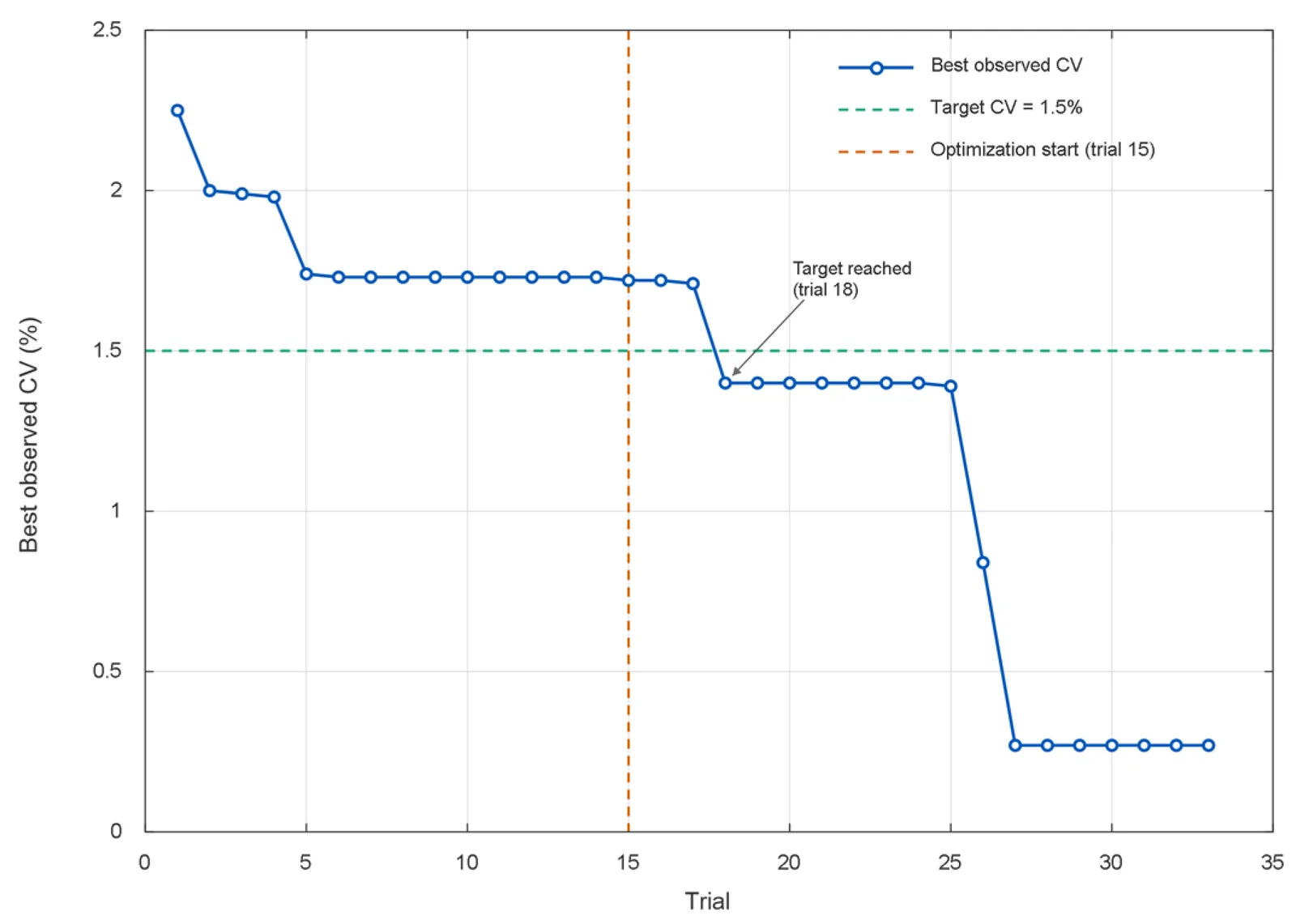
Convergence history of the Bayesian optimization procedure.

**Figure 9:**
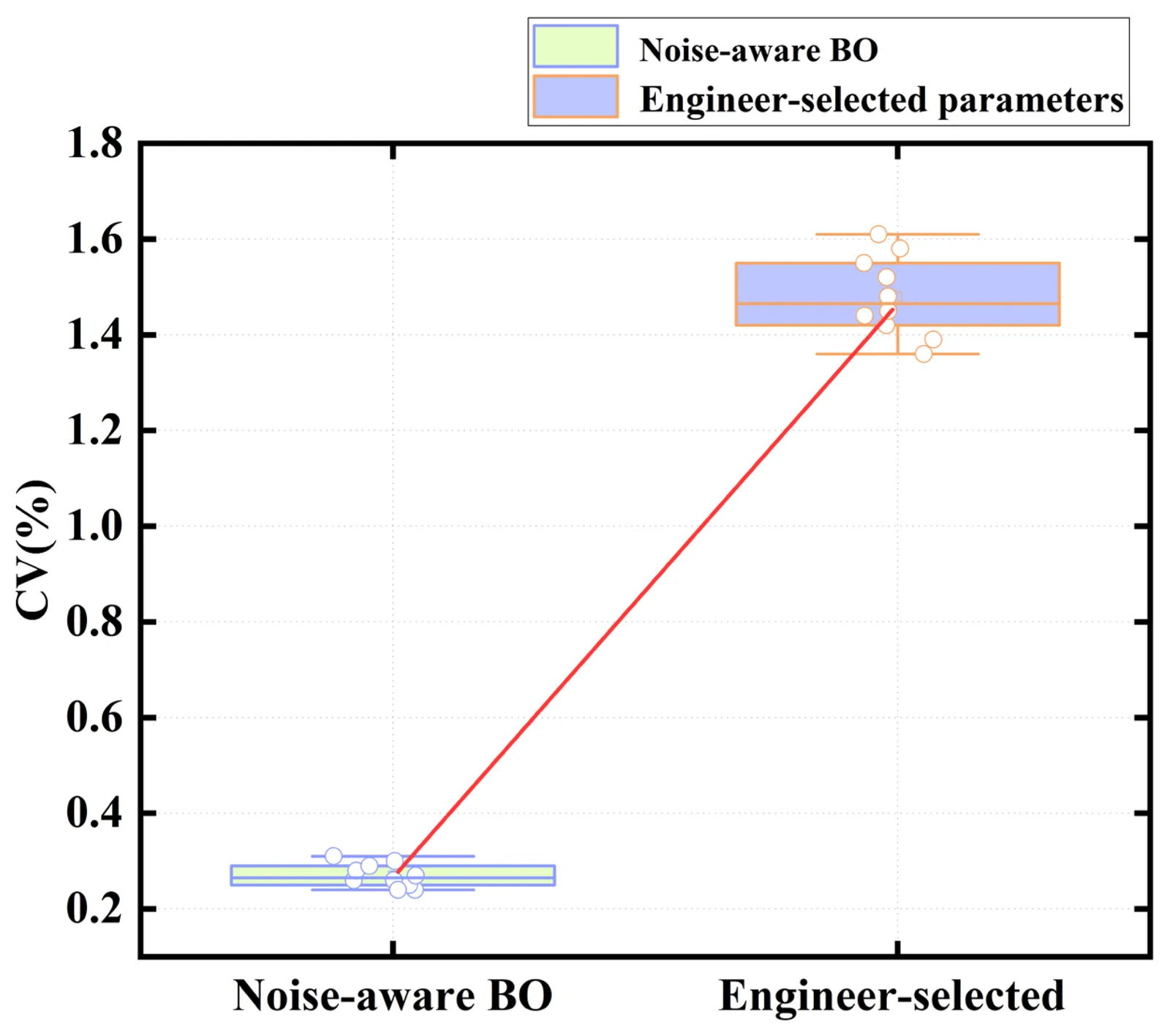
Comparison of the noise-aware Bayesian optimization result with engineer-selected parameters.

**Figure 10:**
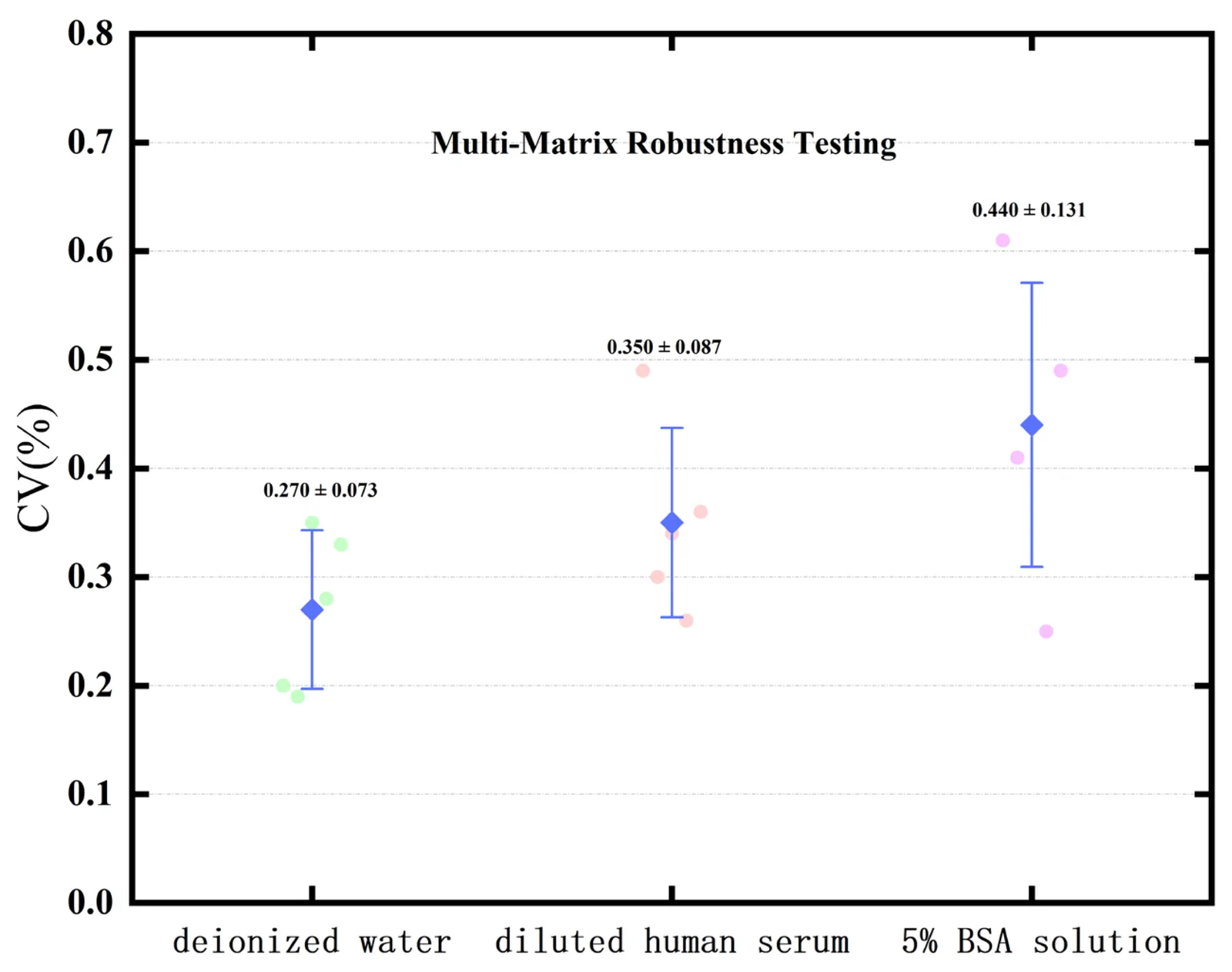
Comparison of dispensing precision across three matrices using the selected parameters.

**Table 1:** Search ranges of the dispensing parameters.

No.	Parameter	Lower Bound	Upper Bound	Unit
1	Dispensing-pump speed	20	150	μL/s
2	Dispensing acceleration	200	3000	μL/s^2^
3	Priming-pump speed	50	400	μL/s
4	Priming acceleration	200	3000	μL/s^2^
5	Aspirated air-column volume	5	50	μL

**Table 2:** Retrospective surrogate-based replay under a shared 18-iteration budget (50 repeats). All strategy trajectories were generated on the same fitted surrogate ground-truth surface and began from the same 15 initial Latin-hypercube points.

Strategy	Iterations to CV < 1.5%	Final Best CV, Mean (%)	Final Best CV, SD (%)	Threshold Reached in >50% of Repeats
Noise-aware GP	2.8 ± 1.2	0.32	0.08	Yes
Homoscedastic GP	4.1 ± 2.0	0.48	0.15	Yes
Standard GP	6.5 ± 3.4	0.61	0.22	Yes
Random search	11.3 ± 5.1	1.22	0.40	No

## Data Availability

The original contributions presented in this study are included in the article. Further inquiries can be directed to the corresponding author.
